# Polygenic Risk Scores and Its Association With Cutaneous Melanoma Risk in the Swedish Population

**DOI:** 10.1111/pcmr.70118

**Published:** 2026-09-17

**Authors:** Hildur Helgadottir, Muyi Yang, Ivette Raices Cruz, Karina Schultz, Veronica Höiom

**Affiliations:** ^1^ Department of Oncology and Pathology Karolinska Institutet Stockholm Sweden; ^2^ Division of Biostatistics, Institute of Environmental Medicine Karolinska Institutet Stockholm Sweden

## Abstract

Polygenic risk scores can quantify an individual's genetic predisposition by combining the effects of multiple gene variants linked to melanoma susceptibility. Previous studies have demonstrated that PRS is strongly associated with melanoma risk and can improve the identification of high‐risk individuals beyond that identified by traditional risk factors. The aim of this study was to calculate PRS for melanoma susceptibility in the Swedish population. Sixty‐four single nucleotide polymorphisms, previously associated with melanoma susceptibility, were used to calculate PRS for 599 melanoma patients with or without family history of melanoma and 1180 controls. PRS values were significantly higher among melanoma patients than among controls, with an area under the receiver operating characteristic curve of 0.710. Individuals in the highest PRS decile had a fourfold increased melanoma risk compared with those in the 5th–6th decile. PRS was strongly influenced by the pigmentation‐related traits skin phototype and hair color, as well as family history of melanoma and, to some extent, the number of primary melanoma tumors. The results show that PRS is strongly associated with melanoma risk in the Swedish population and may serve as a valuable tool for understanding genetic susceptibility and improving risk stratification in melanoma prevention and clinical management.

## Research Highlights

- This is the first study of polygenic risk scores for melanoma in the Swedish population, where incidence is rising, high‐penetrance genes are rarely causal, and risk‐associated phenotypes are common in both patients and the general population.
- There was a strong association between PRS and melanoma risk; individuals with high PRS have a substantially increased melanoma risk compared with those with low scores, suggesting the importance of PRS in genetic risk profiling, especially in familial cases.
- The results also reveal how many small‐effect loci shape melanoma risk beyond that from rare high‐penetrance genes and show clinical potential for identifying high‐risk individuals who may benefit from intensified surveillance and personalized follow‐up.

## Graphical abstract

Established melanoma‐associated genetic variants were genotyped in melanoma patients and population‐based controls from the Swedish population. Polygenic risk scores (PRSs) were subsequently calculated based on the risk effects conferred by these variants. Melanoma patients had significantly higher PRSs than population‐based controls, suggesting that PRSs may have potential utility for improved risk stratification and identification of individuals at increased genetic risk of melanoma.

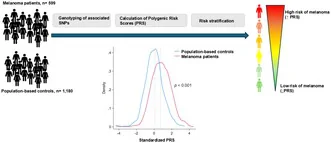

A significant environmental risk factor for cutaneous melanoma (hereafter referred to as melanoma) is intermittent exposure to high levels of ultraviolet (UV) radiation, particularly in individuals with fair skin (Langselius et al. 2025). Over recent decades, melanoma incidence has increased across Europe and other Western countries (Nikolaou and Stratigos 2014; Erdmann et al. 2013), emphasizing the need for improved prevention strategies for this potentially fatal disease.

Melanoma is considered highly heritable, with genetic factors estimated to contribute up to 58% of disease risk (Mucci et al. 2016). About 5%–10% of all melanoma cases occur in families with an inherited predisposition (Olsen et al. 2010). In some families, a pathogenic variant (PV) segregates with the melanoma phenotype in an autosomal dominant manner with high penetrance. The most commonly affected gene is *CDKN2A*. Oher high‐penetrance susceptibility genes include *CDK4, BAP1*, and genes in the telomere‐sheltering complex such as *POT1, TINF2, ACD, TERF2IP*, and *TERT* (Aoude et al. 2015; Helgadottir, Schultz, Lapins, Vassilaki, et al. 2023; Horn et al. 2013; Jensen et al. 2023; Robles‐Espinoza et al. 2014; Walpole et al. 2018; Hoiom et al. 2013; Papadakis et al. 2025). Globally, PVs in high‐penetrance genes explain up to 50% of familial melanoma cases (Read et al. 2016).

In Sweden, fewer than 10% of the melanoma‐prone families carry a known PV (Pissa et al. 2021). In most families without detectable PVs, polygenic inheritance is a more likely explanation for the observed clustering of melanoma within families. Moreover, polygenic factors significantly contribute to melanoma susceptibility among individuals without a family history of the disease.

Genetic profiles associated with melanoma susceptibility are also linked to visible phenotypes such as fair skin, light hair, freckles, and a high number of nevi (Bishop et al. 2009). Variants in the *MC1R* gene, which regulate pigmentation, are well‐established risk alleles for melanoma susceptibility (Palmer et al. 2000). Specific *MC1R* variants, known as red hair color (RHC) variants, are strongly associated with red hair and increased melanoma risk (Goldstein et al. 2005; Höiom et al. 2009; Kennedy et al. 2001). Other *MC1R* variants less strongly linked to red hair (non‐RHC variants) confer a smaller, yet measurable increase in risk (Caini et al. 2020). The *MC1R* gene is highly polymorphic in the Swedish population (Höiom et al. 2009). *MC1R* has been reported to influence melanoma risk through both pigmentation and non‐pigmentation pathways (Tagliabue et al. 2018) and has also been associated with melanoma in individuals lacking the typical high‐risk phenotypes (Kanetsky et al. 2010).

Beyond *MC1R*, genome‐wide association studies (GWAS) have identified numerous low‐penetrance variants independently associated with melanoma susceptibility (Landi et al. 2020). These variants cluster mainly within pathways related to pigmentation, nevus development, and telomere maintenance, although additional pathways—including DNA repair, apoptosis, immunity, melanocyte differentiation, and cell adhesion—have also been implicated.

Individually, most associated gene variants tend to have relatively small effects on the risk. However, by aggregating the influence of multiple variants into a polygenic risk score (PRS), an individual's overall genetic predisposition to melanoma can be estimated. Several studies have demonstrated a strong association between PRS and melanoma risk in other populations (Gu et al. 2018; Pellegrini et al. 2024; Potjer et al. 2021; Steinberg et al. 2022; Cust et al. 2018; Law et al. 2020), but such analysis has not been assessed earlier in any Nordic population. The Nordic countries, in particular Sweden, Norway, and Denmark have, after Australia and New Zealand, the highest melanoma incidences globally (International Agency for Research on Cancer of the World Health Organization. Global Cancer Observatory, Global Cancer Observatory). Pigmentation traits and sun‐seeking behaviors likely have a strong influence on melanoma risk in the Nordic area (Jerkegren et al. 1999; Johansen et al. 2016).

In this study, we combined melanoma‐associated variants into a PRS to assess genetic risk in the Swedish population. The aim was to explore if PRS could aid in melanoma risk stratification by detecting individuals with unfavorable genetic risk profiles that might benefit from targeted prevention programs.

The patient cohort included 599 melanoma cases followed at Karolinska University Hospital, Stockholm, between 1993 and 2018, subdivided into single primary melanoma without family history of melanoma (SPM, *n* = 244), multiple primary melanomas without family history of melanoma (MPM, *n* = 211), and familial melanoma (FM, *n* = 144). Familial cases were defined as patients from families with either three melanoma cases or two first‐degree relatives if one was diagnosed before age 55. All cases were unrelated and negative for PVs in *CDKN2A*. As comparison, we used controls from the general population; one control cohort of 180 blood donors from Stockholm where data on sex, hair color, and skin type were available (Höiom et al. 2009), as well as genotype data from SweGen that contain genome variant data from 1000 Swedish individuals (Ameur et al. 2017), representing a cross‐section of the Swedish population. Characteristics of the melanoma cases and controls included in the study are shown in Table 1.

**TABLE 1:** Characteristics of the control and melanoma population included in the study with corresponding standardized polygenic risk scores (sPRS), *p*‐values from Mann–Whitney test or Kruskall–Wallis test.

	Controls (SweGen)	Controls (in house)	All melanoma cases	Single sporadic melanoma	Multiple sporadic melanoma	Familial melanoma cases	*p*
	*N* = 1000	*N* = 180	*N* = 599	*N* = 244	*N* = 211	*N* = 144	
Sex, *N* (%)							
Male	506 (51)	87 (48)	285 (48)	119 (49)	102 (48)	64 (45)	0.301
Female	494 (49)	93 (52)	313 (52)	125 (51)	109 (52)	79 (55)	
Age at melanoma diagnosis							
Mean/median (range)	NA	NA	52.5/54.0 (13–88)	50.2/53.0 (13–86)	60.0/62.0 (16–88)	45.5/43.0 (15–83)	NA
Hair color, *N* (%)							
Red	N/A	10 (6)	61 (11)	18 (8)	25 (12)	18 (18)	< 0.001
Light blond	N/A	27 (15)	123 (23)	45 (19)	56 (27)	22 (22)	
Medium Blond	N/A	90 (50)	282 (52)	142 (61)	91 (43)	49 (50)	
Brown	N/A	53 (29)	77 (14)	29 (12)	38 (18)	10 (10)	
Fitzpatrick phototype, *N* (%)							
I	N/A	1 (1)	64 (11)	20 (8)	29 (14)	15 (13)	< 0.001
II	N/A	28 (16)	200 (36)	75 (32)	78 (37)	47 (41)	
III	N/A	126 (70)	266 (48)	123 (52)	91 (44)	52 (46)	
IV	N/A	25 (14)	29 (5)	18 (8)	11 (5)	0 (0)	
Number of primary melanomas							
Mean/median (range)	N/A	0.0/0.0 (0–0)	1.7/1.0 (1–16)	1.0/1.0 (1–1)	2.5/2.0 (2–16)	1.6/1.0 (1–8)	NA
Standardized PRS (sPRS)							
Mean (SD) Median (Q1, Q3)	0.0 (1.00) −0.015 (−0.647, 0.572)		0.787 (1.116) 0.742 (0.035, 1.513)	0.621 (1.093) 0.613 (−0.142, 1.374)	0.812 (1.173) 0.874 (0.133, 1.514)	1.032 (1.023) 0.981 (0.375, 1.733)	< 0.001

*Note:* *p‐*value in all melanoma cases versus in house controls for sex, hair color (red‐light or blond vs. dark‐blond or brown) and Fitzpatrick phototype (1–II vs. III–IV) using Chi‐squared test.

Abbreviations: N/A, not available; NA, not applicable; Q1, the first quartile—25th percentile of the data; Q3, third quartile—75th percentile of the data; SD, standard deviation.

The PRS for every individual was calculated by combining genotype‐specific relative risks for 64 SNPs in a log‐additive model. Information about the included gene variants, with nearest gene, odds ratios (ORs), and allele frequencies, is found in Table S1.

For comparison purposes between cases and control, the PRS was standardized (sPRS) to the mean and SD in the control cohort. A detailed description of patient accrual and characteristics, SNP selection and genotyping, calculation of PRS, and statistical analyses is available in Supporting Information. Informed consent was attained from all patients, and the study was approved by the Swedish Ethical Review Authority.

Melanoma patients exhibited significantly higher sPRS than controls (mean 0.787, SD = 1.116), with more than three‐quarters (76%) of patients having scores above the population mean (Table 1 and Figure 1). This is in line with previous reports from other populations (Pellegrini et al. 2024; Potjer et al. 2021; Gu et al. 2018), reflecting the increased burden of common genetic variants among melanoma patients.

**FIGURE 1:**
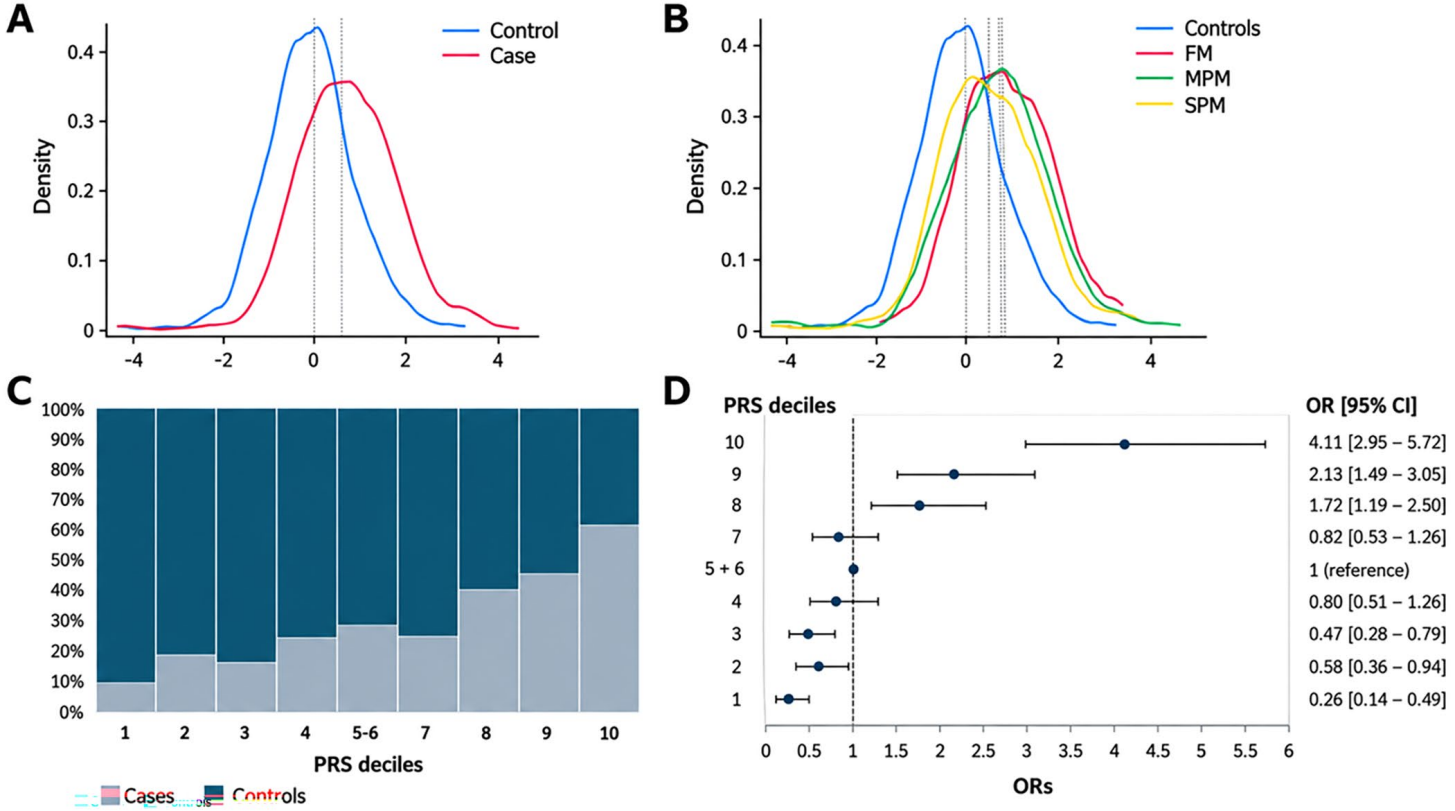
Melanoma risk is significantly associated with standardized PRS. (A) Distribution of sPRS in all cases (*n* = 599) and controls (*n* = 1180), and in (B) controls and cases divided in familial melanoma (FM), sporadic primary melanoma (SPM), and sporadic multiple primary melanomas (MPM). (C) The proportion of melanoma cases and controls assigned to different deciles. Melanoma cases were categorized based on the sPRS distribution established by the control group. (D) Odds ratios (ORs) and 95% confidence intervals (CI) for melanoma risk by sPRS decile compared to the 5–6th decile. Points represent estimated ORs and the vertical lines denote the corresponding 95% CI. The dashed line indicates OR = 1.

Stratification by clinical subgroup revealed a gradient of increasing genetic burden, with the highest mean sPRS in FM cases (1.032, SD = 1.023), followed by MPM (0.812, SD = 1.173) and SPM (0.621, SD = 1.093) (Table 1). All subgroups differed significantly from controls. In a study by Law et al. (2020), significant higher mean polygenic load for familial melanoma cases compared to unrelated cases and controls were reported. We could see a significant difference in sPRS between FM and SPM (mean sPRS 1.032 vs. 0.621, *t*‐test *p* < 0.001) or MPMs with two to three primary melanomas (mean sPRS 1.032 vs. 0.760, *t*‐test *p* = 0.028), whereas there was no significant difference between FM and MPMs with 4 or more tumors (mean sPRS 1.032 vs. 1.171). This indicates that patients with many primary melanomas also are enriched for genetic risk as the familial cases (also classified as “familial” in the Swedish national guidelines for familial melanoma). Similar observations have been reported beyond melanoma, for example familial breast cancer patients with bilateral breast cancer has been demonstrated to exhibit significantly higher PRS values than patients with unilateral breast cancer (Kvist et al. 2025).

Logistic regression analysis further quantified the association between PRS and melanoma risk (Figure S1). Each unit increase in sPRS was associated with more than a twofold increase in melanoma risk (OR = 2.09, 95% CI 1.88–2.33, *p* < 0.001). Importantly, this association remained robust after adjustment for pigmentation traits (adjusted OR = 1.76, 95% CI 1.47–2.10, *p* < 0.001), indicating that PRS captures risk beyond traditional phenotypic markers. Subgroup analyses showed significant associations across all melanoma categories, with the strongest effect observed in FM (adjusted OR = 2.74, 95% CI 1.95–3.85, *p* < 0.001).

The discriminative performance of sPRS was evaluated using AUC analysis (Figure 2). The sPRS‐only model achieved an AUC of 0.724 (95% CI 0.682–0.766), slightly outperforming the pigmentation model (AUC = 0.688, 95% CI 0.644–0.731). Combining PRS with pigmentation traits significantly improved discrimination (AUC = 0.751, 95% CI 0.711–0.792), corresponding to a 6.3% increase in predictive performance (Table S2). This was even more pronounced when analyzing FM and cases without family history separately: the combined model reached an AUC of 0.826 (8.8% improvement) and 0.736 (5.8% improvement) for those with a family history and those without family history, respectively. This is higher than shown, for example in the study by Cust et al. (2018) with a 2.3%–2.8% improvement in risk prediction by adding PRS to pigmentation traits. However, compared to our study they had more risk factors included in their traditional model, as well as fewer SNPs included in the PRS. Notably, PRS alone discriminated significantly better than pigmentation traits among FM cases (*p* = 0.039), further emphasizing the importance of polygenic risk in this subgroup. The AUC of 0.706 for our cases without family history was similar to those described in cohorts from the UK and Australia (Steinberg et al. 2022) but somewhat higher than another 68‐SNP PRS based on UK Biobank that had an estimated AUC of 0.639 (Wong et al. 2023).

**FIGURE 2:**
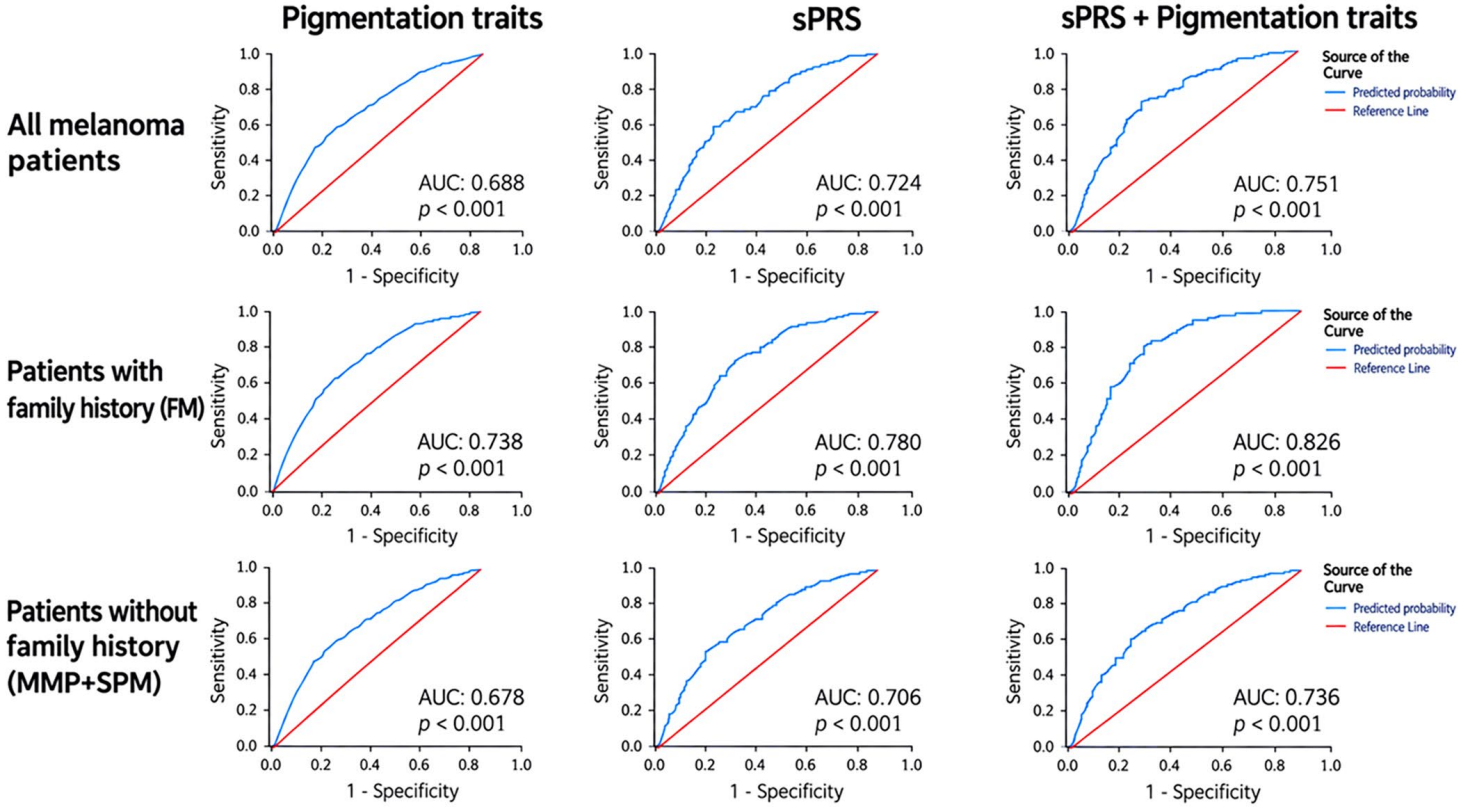
The area under the ROC curve (AUC) was used to evaluate the performance of PRS and pigmentation traits (hair color and Fitzpatrick phototype) as prediction models for melanoma risk. The analysis was done as univariate models or as a combined model. Patients were analysed all together or separate based on the presence of family history or not (sporadic cases, both those with single and multiple primary melanomas). Only the in‐house controls (where phenotype data was available) were included in this analysis.

Risk stratification by PRS deciles (based on the control distribution) revealed a strong dose–response relationship between genetic risk and melanoma prevalence (Figure 1C,D). Individuals in the highest decile had nearly a fourfold increased risk compared with those in the middle deciles (OR = 3.88, 95% CI 2.04–7.35), while those in the lowest decile had a substantially reduced risk (OR = 0.24, 95% CI 0.18–0.34). This gradient highlights the potential clinical utility of PRS for identifying individuals at particularly high or low risk within the population.

Further analyses examined the relationship between PRS and patient characteristics as well as familial aggregation. Across all melanoma cases, mean sPRS was significantly associated with hair color, phototype, and family history, but not with sex, age at onset, or number of primary melanomas (Table S3). Several studies have previously demonstrated a correlation between higher PRS and the occurrence of multiple primary melanomas (Olsen et al. 2023; Pellegrini et al. 2024; Potjer et al. 2021). However, when restricting the analysis to SPM and MPM only, a modest but significant association between sPRS and number of primary melanomas emerged (*p* = 0.044).

Within FM cases, multiple linear regression identified the number of melanomas per family as the only significant predictor of mean sPRS (*p* = 0.003) (Table S4). A clear trend was observed, with mean sPRS increasing from 0.834 in families with two affected members to 1.810 in families with six or more melanomas (Table S5). This finding contrasts with that of Potjer et al. (2021), who reported higher PRS in low‐density families and lower PRS in high‐density families, implying the presence of an unidentified high‐risk genetic factor and a less significant role of PRS in their high‐density families, whereas our results suggest a significant role for PRS, especially for the Swedish melanoma‐dense families. In studies on familial breast cancer patients, increased PRS has been observed both among non‐carriers and carriers of moderate‐ and high‐penetrance PVs, suggesting that polygenic background may influence cancer risk in both groups (Kvist et al. 2025; Li et al. 2017). In a previous report from our group, a significant factor when estimating the relative risk for new melanomas in *CDKN2A*‐negative melanoma families was the presence of at least one cutaneous SCC in the family (Helgadottir, Schultz, Lapins, and Hoiom 2023). Here, we could not see that the presence of cSCC in the family was a predictor of PRS.

In line with established epidemiological patterns, melanoma patients showed significantly higher frequencies of red or light blond hair and Fitzpatrick phototype I–II compared with controls (both *p* < 0.001), confirming the strong contribution of pigmentation‐related susceptibility for melanoma in Sweden. When analysing pigmentation characteristics across the combined dataset of patients and controls (Table S6), sPRS demonstrated a clear and statistically significant association with pigmentation traits. Individuals with sun‐sensitive skin (Fitzpatrick phototype I–II) had higher sPRS than those with more protective phototypes (III–IV). A gradient was also evident across hair color, with the highest sPRS observed among red‐haired individuals, followed by light blond, blond, and lastly brown/dark hair. Multiple regression analysis confirmed that both phototype and hair color independently predicted sPRS, confirming that PRS captures genetic variation closely tied to visible phenotypic risk factors. Eye color has also been proposed as a phenotypic risk factor for melanoma. For example, the rs12913832 variant in *HERC2* (included in this and many other PRS models) has been associated with both blue eye color and an increased risk of melanoma (Calbet‐Llopart et al. 2022). In our Swedish cohort, however, rs12913832 was highly prevalent, with similar variant allele frequencies in controls (88%) and melanoma cases (90%). In Sweden, blue eyes are common and any minor effect on melanoma risk disappears after adjusting for other pigmentation characteristics, like skin type. Eye color was therefore not considered in this study.

However, PRS was not solely a proxy for pigmentation phenotype, but also identified high‐risk individuals without the classic risk phenotype. In our study, 102 melanoma patients had a less sensitive phototype and dark (brown) hair color, and of these 19 (18%) had a sPRS in the top 20%, indicating that PRS captured melanoma risk beyond that of traditional pigmentation factors.

Taken together, these findings provide the first comprehensive evaluation of PRS in a Nordic melanoma population and demonstrate that polygenic risk plays a substantial role in melanoma susceptibility in this high‐incidence region.

The observed associations between PRS, pigmentation traits, and melanoma risk are consistent with prior studies conducted in non‐Nordic populations, reinforcing the biological validity of PRS. The results also support the concept of two complementary genetic pathways in melanoma development: one driven by rare, high‐penetrance variants and another driven by the cumulative effect of multiple common variants captured by PRS. In this cohort, which excluded carriers of pathogenic *CDKN2A* variants, the most commonly known high penetrance genetic factor for melanoma, polygenic inheritance appears to play a dominant role, particularly in families with multiple affected individuals or in patients with many primary melanomas.

Although our findings are based on data from one region, Stockholm's demographic composition—shaped by urbanization and continuous migration from various regions across Sweden—gives a study cohort that in many ways reflects the general Swedish population, thereby supporting the broader national relevance of the findings.

Some limitations with the study should be considered. The lack of comprehensive pigmentation data among a majority of the controls and absence of information on UV exposure, sun behavior, and nevus phenotype may have influenced the estimates of risk and model performance. In addition, mutation status was only available for *CDKN2A* in our patients. However, pathogenic variants (PVs) in other high‐risk genes, such as *BAP1* and telomere‐related genes, are considered rare and are estimated to account for only 2%–3% of FM cases in Sweden (Helgadottir, Schultz, Lapins, Vassilaki, et al. 2023; Papadakis et al. 2025), they are also believed to be exceedingly uncommon among patients without a family history of melanoma.

Our results show that PRS can distinguish melanoma risk within the Swedish population and may help stratify individuals into high‐, medium‐, and low‐risk groups, potentially guiding inclusion in preventive programs. However, further studies are needed before clinical implementation; particularly it would be important to study the synergy with UV exposure. It is also important to note that PRS reflects relative rather than absolute risk—individuals with high PRS may never develop melanoma, while those with low PRS may still be affected.

## Author Contributions

**Hildur Helgadottir:** investigation, writing – review and editing, resources, data curation, conceptualization, funding acquisition. **Muyi Yang:** investigation, methodology, writing – review and editing. **Ivette Raices Cruz:** writing – review and editing, methodology, formal analysis, visualization. **Karina Schultz:** writing – review and editing, data curation, investigation. **Veronica Höiom:** conceptualization, funding acquisition, writing – original draft, data curation, resources, investigation, formal analysis, visualization, methodology, supervision.

## Funding

This work was supported by Cancerfonden (20 0156 F, 21 1486 Pj, and 24 3826 Pj), Region Stockholm (FoUI‐975235 and FoUI‐1023897), Radiumhemmets Forskningsfonder (194092, 2240233, and 194103), Stiftelsen Tornspiran.

## Ethics Statement

The study was conducted in accordance with Good Clinical Practice with informed consent from all patients and was approved by the Stockholm Regional Ethics Committee (Dnr: 03‐471, amendments 2012/1192‐32 and 2025‐05143‐02).

## Conflicts of Interest

The authors declare no conflicts of interest.

## Supporting information


**Figure S1:** Odds ratios (ORs) for sPRS in melanoma cases compared to controls, as univariate logistic regression or adjusted for hair color and skin type.
**Table S1:** Overview of SNPs included in the Polygenic risk scores (PRS). Odds ratios (OR) per effect allele are taken from previous reported meta‐GWAS (Landi et al. 2020).
**Table S2:** Comparison between the discrimination ability of the different models (univariate PRS, univariate pigmentation traits, combined model) using paired‐sample AUC.
**Table S3:** Polygenic risk score (PRS) distribution by both familial and sporadic patient characteristics. *p*‐value from Mann–Whitney test or Kruskall–Wallis test.
**Table S4:** How different family features predict sPRS (events occurring in the family, yes or no) using multiple regression analysis.
**Table S5:** Standardized polygenic risk score (sPRS) distribution in dependent on number of melanoma cases in a family. *p*‐values from Kruskall–Wallis test on independent samples.
**Table S6:** Standardized polygenic risk score (sPRS) distribution in patient and control individuals (combined) by characteristics. *p*‐values from Mann–Whitney test or Kruskall–Wallis test.

## Data Availability

The data that support the findings of this study are available from the corresponding author upon reasonable request.
