# Supplementary material for: Polygenic Risk Scores and Its Association With Cutaneous Melanoma Risk in the Swedish Population
Source: Pigment Cell Melanoma Res. 2026 Sep 17;39(5):e70118. doi: 10.1111/pcmr.70118 (PMC13586051; doi:10.1111/pcmr.70118)
Supplement: Supplementary file 1 [file PCMR-39-0-s001.docx]

**Supplementary Methods**

**Study population**

The patient cohort included melanoma cases followed at Karolinska University Hospital, Stockholm, between 1993 and 2018. Familial cases were defined as patients from families with either three melanoma cases or two first-degree relatives if one was diagnosed before age 55. All cases were unrelated. Patients with at least two independent primary melanomas and no family history formed the sporadic multiple primary group, while a third group included sporadic single primary cases. Hair color (red, light blond, medium blond or brown/dark) and skin type (Fitzpatrick phototype I–II vs. phototype III–IV) were extracted from medical records. All patients have been screened for PVs in *CDKN2A* with negative results.

The control data set consisted partly of 180 blood donors from Stockholm (Höiom et al. 2009). To qualify as a blood donor you must be healthy, not have an active cancer diagnosis or a history of cancer. Data on sex as well as for hair color and skin type were available for this cohort.

A second control data set consisted of the SweGen controls. This data set contains genome variant data from 1,000 Swedish individuals (Ameur et al. 2017). This cohort represents a cross-section of the Swedish population; thus, no disease status of included individuals is available. Genotype data for the selected SNPs (see below) were extracted from UPPMAX (Uppsala Multidisciplinary Center for Advanced Computational Science) through the National Academic Infrastructure for Supercomputing in Sweden (NAISS) - Sensitive data (SENS) project.

**SNP selection and genotyping**

The SNPs were chosen from the previously published PRS68-panel, based on their validated associations with melanoma (Landi et al. 2020). Genotyping was done at the Translational Analysis in Molecular Medicine (TAMM) core facility at Karolinska Institutet. The method used was performed on a MassARRAY Platform from Agena ([www.agenabio.com](http://www.agenabio.com)), using an iPLEX chemistry allowing simultaneously genotyping of multiple SNPs. When designing the pools, 8 SNPs failed, of which five were successfully replaced. In addition, two *MC1R* variants were included, leaving 67 SNP assays divided into three pools. Three SNPs failed the quality controls. Success rate for remaining SNPs was over 98, 5% and the accuracy of calls was > 99.7 %.

**Polygenic risk scores**

The risk for each variant was based on allele frequencies and effect sizes (ORs). The ORs were estimates from the previously reported melanoma meta-GWAS (Landi et al. 2020). Genotype-specific relative risks for each gene variant were determined as previously been described (Mealiffe et al. 2010; Steinberg et al. 2022). In summary, genotype-specific relative risks for each variant were calculated as follows: SNP with genotypes AA, AB, and BB and an odds ratio *OR_SNP_* for allele B vs allele A has corresponding genotype-specific relative risks of 1, *OR_SNP_*, and *OR^2^_SNP_*. If the frequency of allele B in the population is *p*_SNP_, then under Hardy-Weinberg equilibrium, the frequencies of the genotypes AA, AB, and BB are (1− *p*_SNP_,)^2^, 2*p_SNP_*, (1 - *p*_SNP_), and *p*_SNP_^2^(1−pSNP), respectively. The expected average relative risk in the population is therefore: μ_SNP_ = (1 − *p*_SNP_)^2^ + 2*p*_SNP_(1 − *p*_SNP_)OR_SNP_ + *p*_SNP_^2^OR_SNP_^2^. The genotype-specific relative risks for each SNP were then normalised by μ so that the expected average relative risk in the population would be 1, i.e. used the scaled relative risks 1/μ_SNP_, OR_SNP_/μ_SNP_, and OR_SNP_^2^/μ_SNP_ for AA, AB, and BB genotypes, respectively. The PRS for every participant were then calculated by combining the genotype-specific relative risks for specific SNPs in a log-additive model. Any missing genotypes were replaced with the μSNP value for each SNP according to the formula above.

. The PRS for every participant were thereafter calculated by combining the genotype-specific relative risks for all SNPs in a log-additive model. For comparison purposes between cases and control, the PRS was standardized (sPRS) to the mean and SD in the control cohort giving a sPRS mean of 0 with an SD of 1 in the controls.

**Statistical Methods**

Association between PRS and melanoma risk was analyzed using binary logistic regression and adjusted for skin type, hair color and sex. Association with melanoma risk using sPRS as a categorical variable was also evaluating by using deciles of controls as cut-off points groups, considering the 5–6th as references. The Mann-Whitney test was applied for comparing distribution of the sPRS between two independent groups whereas Kruskall–Wallis test was used when comparing three or more groups. The discriminative ability of the PRS model, a pigmentation model (hair and skin-type) and a combined model (sPRS+ pigmentation) was assessed with Area Under the receiver-operating Curve (AUC) analysis. Comparison between models was evaluated using paired-sample AUC. Predictors of PRS was evaluated using multivariable linear regression considering both patient characteristics and family features for familial cases. All statistical analyses were performed using Stata 17 or IBM SPSS v30 Statistics.

**REFERENCES**

Ameur, A., J. Dahlberg, P. Olason, et al. 2017. 'SweGen: a whole-genome data resource of genetic variability in a cross-section of the Swedish population', *Eur J Hum Genet*, 25: 1253-60.

Höiom, V., R. Tuominen, M. Kaller, et al. 2009. 'MC1R variation and melanoma risk in the Swedish population in relation to clinical and pathological parameters', *Pigment Cell Melanoma Res*, 22: 196-204.

Landi, M. T., D. T. Bishop, S. MacGregor, et al. 2020. 'Genome-wide association meta-analyses combining multiple risk phenotypes provide insights into the genetic architecture of cutaneous melanoma susceptibility', *Nat Genet*, 52: 494-504.

Mealiffe, M. E., R. P. Stokowski, B. K. Rhees, et al. 2010. 'Assessment of clinical validity of a breast cancer risk model combining genetic and clinical information', *J Natl Cancer Inst*, 102: 1618-27.

Steinberg, J., M. M. Iles, J. Y. Lee, et al. 2022. 'Independent evaluation of melanoma polygenic risk scores in UK and Australian prospective cohorts', *Br J Dermatol*, 186: 823-34.

**Supplementary figures**

**Supplementary figure 1.** Odds ratios (ORs) for sPRS in melanoma cases compared to controls, as univariate logistic regression or adjusted for hair color and skin type.

**Supplementary tables**

**Supplementary Table S1**. Overview of SNPs included in the Polygenic risk scores (PRS). Odds ratios (OR) per effect allele are taken from previous reported meta-GWAS (Landi et al. 2020)

| **rsID** | **Reference allele (effect allele)** | **Variant allele** | **Chromosome** | **Position (GRCh38)** | **OR (per allele EA)** | **Effect allele frequency (In house ctrls + SweFreq)** | **None-effect allele frequency (In house ctrls + SweFreq)** | **None-effect allele frequency cases** | **Nearest gene** |
| --- | --- | --- | --- | --- | --- | --- | --- | --- | --- |
| rs670318 | T | C | chr1 | 63261871 | 0,8646 | 0,054422 | 0,945578 | 0,954 | *LINC00466* |
| rs8444 | G | A | chr1 | 150966095 | 1,0801 | 0,662692 | 0,337308 | 0,321 | *RP11-316M1.12* |
| rs76798800 | G | T | chr1 | 155022502 | 0,9174 | 0,887564 | 0,112436 | 0,293 | *DCST2* |
| rs2369633 | T | C | chr1 | 205211934 | 1,0951 | 0,075319 | 0,924681 | 0,91 | *DSTYK* |
| rs2695237 | T | C | chr1 | 226415934 | 1,0947 | 0,612766 | 0,387234 | 0,35 | *RP11-118H4.3* |
| rs7902587 | C | T | chr10 | 103934543 | 0,8563 | 0,91284 | 0,08716 | 0,107 | *OBFC1* |
| rs7941496 | G | T | chr11 | 16019759 | 1,0602 | 0,556644 | 0,443356 | 0,435 | *CTD-3096P4.1* |
| rs4354713 | A | G | chr11 | 69566130 | 1,0969 | 0,32155 | 0,67845 | 0,662 | *CCND1* |
| rs1126809 | G | A | chr11 | 89284793 | 0,8272 | 0,733844 | 0,266156 | 0,301 | *TYR* |
| rs1801516 | G | A | chr11 | 108304735 | 1,1426 | 0,850683 | 0,149317 | 0,14 | *ATM* |
| rs12290699 | T | C | chr11 | 120324993 | 0,9379 | 0,764456 | 0,235544 | 0,224 | *TMEM136* |
| rs1056927 | A | G | chr12 | 12917818 | 0,9257 | 0,604592 | 0,395408 | 0,409 | *MIR614* |
| rs4237963 | T | A | chr12 | 17122526 | 0,9276 | 0,20017 | 0,79983 | 0,815 | *RPL7P40* |
| rs1278768 | G | C | chr13 | 112881635 | 0,9358 | 0,478687 | 0,521313 | 0,562 | *MCF2L* |
| rs12913832 | A | G | chr15 | 28120472 | 0,8839 | 0,121071 | 0,878929 | 0,902 | *HERC2* |
| rs117648907 | C | T | chr15 | 32985509 | 0,8039 | 0,981277 | 0,018723 | 0,022 | *SNORD77* |
| rs4420522 | A | G | chr16 | 68789068 | 0,9259 | 0,723169 | 0,276831 | 0,327 | *CDH1* |
| rs2967383 | G | T | chr16 | 82183548 | 1,0649 | 0,320851 | 0,679149 | 0,685 | *RN7SKP190* |
| rs116927526 | C | T | chr16 | 89677219 | 0,5934 | 0,997449 | 0,002551 | 0,01 | *CDK10* |
| rs1805006 | C | A | chr16 | 89919510 | 0,68 | 0,992347 | 0,007653 | 0,01 | *MC1R* |
| rs11547464 | G | A | chr16 | 89919683 | 0,68 | 0,993628 | 0,006372 | 0,008 | *MC1R* |
| rs1805007 | C | T | chr16 | 89919709 | 0,574 | 0,920851 | 0,079149 | 0,138 | *MC1R* |
| rs1805008 | C | T | chr16 | 89919736 | 0,7307 | 0,889881 | 0,110119 | 0,151 | *MC1R* |
| rs1805009 | G | C | chr16 | 89920138 | 0,6777 | 0,994053 | 0,005947 | 0,015 | *MC1R* |
| rs3212371 | A | G | chr16 | 89920793 | 0,9186 | 0,906383 | 0,093617 | 0,102 | *TUBB3* |
| rs78378222 | T | G | chr17 | 7668434 | 0,7568 | 0,983844 | 0,016156 | 0,029 | *TP53* |
| rs12608592 | C | T | chr19 | 3542985 | 0,6531 | 0,977466 | 0,022534 | 0,028 | *MFSD12* |
| rs61712781 | T | C | chr2 | 25556626 | 0,9332 | 0,771259 | 0,228741 | 0,246 | *DTNB* |
| rs1800440 | T | C | chr2 | 38070996 | 1,1018 | 0,829652 | 0,170348 | 0,151 | *CYP1B1* |
| rs10931936 | T | C | chr2 | 201279205 | 1,0813 | 0,254036 | 0,745964 | 0,692 | *CASP8* |
| rs6059655 | A | G | chr20 | 34077942 | 1,4481 | 0,018282 | 0,981718 | 0,897 | *RALY* |
| rs143190905 | G | T | chr20 | 63660414 | 1,1454 | 0,923895 | 0,076105 | 0,069 | *RTEL1* |
| rs408825 | C | T | chr21 | 41371569 | 0,8889 | 0,398384 | 0,601616 | 0,69 | *MX2* |
| rs132985 | C | T | chr22 | 38167464 | 1,12 | 0,571612 | 0,428388 | 0,383 | *PLA2G6* |
| rs5766565 | A | G | chr22 | 45226803 | 1,0632 | 0,65051 | 0,34949 | 0,329 | *KIAA0930* |
| rs79966207 | T | C | chr22 | 50283979 | 0,9238 | 0,829082 | 0,170918 | 0,194 | *PLXNB2* |
| rs149617956 | G | A | chr3 | 69964940 | 0,3847 | 0,99915 | 0,00085 | 0,003 | *MITF* |
| rs3950296 | C | G | chr3 | 169775495 | 1,0775 | 0,73852 | 0,26148 | 0,23 | *MYNN* |
| rs7726159 | C | A | chr5 | 1282204 | 0,916 | 0,6546 | 0,3454 | 0,376 | *TERT* |
| rs2853667 | A | G | chr5 | 1299955 | 1,0623 | 0,601536 | 0,398464 | 0,379 | *TERT* |
| rs13178866 | C | T | chr5 | 1323097 | 0,8672 | 0,51234 | 0,48766 | 0,514 | *CLPTM1L* |
| rs16891982 | C | G | chr5 | 33951588 | 0,5046 | 0,028462 | 0,971538 | 0,991 | *SLC45A2* |
| rs12523094 | T | C | chr5 | 90966795 | 1,0654 | 0,58801 | 0,41199 | 0,35 | *CTD-2266L18.1* |
| rs32578 | G | A | chr5 | 149832305 | 1,0903 | 0,645408 | 0,354592 | 0,361 | *PPARGC1B* |
| rs12215602 | G | A | chr6 | 1145030 | 0,9414 | 0,71131 | 0,28869 | 0,301 | *snoU13* |
| rs72834823 | T | A | chr6 | 22719150 | 1,0972 | 0,822828 | 0,177172 | 0,158 | *RP1-309H15.2* |
| rs28986343 | C | T | chr6 | 32781176 | 1,1531 | 0,931005 | 0,068995 | 0 | *HLA-DQB2* |
| rs73069846 | C | T | chr7 | 16956904 | 0,9457 | 0,597353 | 0,402647 | 0,393 | *AGR3* |
| rs117132860 | G | A | chr7 | 17095084 | 0,7114 | 0,991489 | 0,008511 | 0,02 | *AHR* |
| rs12539524 | C | T | chr7 | 22075836 | 0,9264 | 0,822128 | 0,177872 | 0,171 | *RAPGEF5* |
| rs4731207 | G | A | chr7 | 124756591 | 0,9265 | 0,536139 | 0,463861 | 0,51 | *GPR37* |
| rs7778378 | C | T | chr7 | 131053907 | 0,9336 | 0,218537 | 0,781463 | 0,831 | *MKLN1* |
| rs6994183 | A | T | chr8 | 22093498 | 0,9222 | 0,883491 | 0,116509 | 0,127 | *FAM160B2* |
| rs13263376 | G | A | chr8 | 71952005 | 0,9283 | 0,33716 | 0,66284 | 0,688 | *RP11-383H13.1* |
| rs10960710 | G | T | chr9 | 12587153 | 0,9322 | 0,308085 | 0,691915 | 0,695 | *TYRP1* |
| rs871024 | C | A | chr9 | 21803881 | 1,1794 | 0,468963 | 0,531037 | 0,462 | *MTAP* |
| rs2188127 | C | G | chr9 | 21965233 | 1,09 | 0,892007 | 0,107993 | 0,099 | *CDKN2A* |
| rs55797833 | T | G | chr9 | 21995045 | 1,5478 | 0,981293 | 0,018707 | 0,01 | *CDKN2B-AS1* |
| rs3217986 | T | G | chr9 | 22005331 | 0,8532 | 0,931973 | 0,068027 | 0,108 | *CDKN2B* |
| rs79356439 | A | G | chr9 | 22011361 | 1,8504 | 0,991497 | 0,008503 | 0,001 | *UBA52P6* |
| rs75883022 | C | T | chr9 | 22067911 | 0,7927 | 0,979574 | 0,020426 | 0,025 | *CDKN2B-AS1* |
| rs10739220 | C | T | chr9 | 106292136 | 1,1036 | 0,255952 | 0,744048 | 0,734 | *RP11-308N19.1* |
| rs1339759 | C | G | chr9 | 107949305 | 1,0946 | 0,587086 | 0,412914 | 0,381 | *RPS15AP27* |
| rs3780269 | G | A | chr9 | 131582193 | 0,9443 | 0,645408 | 0,354592 | 0,368 | *RAPGEF1* |

**Supplementary Table S2.** Comparison between the discrimination ability of the different models (univariate PRS, univariate pigmentation traits, combined model) using paired-sample AUC.

| **Patient cohort** | **Test results pairs** | **Z** | ***p*-value** | **AUC difference** | **95 % CI** |
| --- | --- | --- | --- | --- | --- |
| **All patients** | Pigmentation vs PRS | 1.494 | 0.135 | -0.036 | -0.011 – 0.083 |
|  | Pigmentation vs Combination | 4.204 | <0.000 | 0.063 | 0.034 - 0.093 |
|  | PRS vs Combination | 2.231 | 0.026 | 0.027 | 0.003 – 0.052 |
| **Familial cases** | Pigmentation vs PRS | 2.061 | 0.039 | -0.067 | 0.003 – 0.131 |
|  | Pigmentation vs Combination | 3.684 | <0.001 | 0.088 | 0.041 – 0.135 |
|  | PRS vs Combination | 1.786 | 0.074 | 0.021 | -0.002 – 0.044 |
| **Sporadic cases** | Pigmentation vs PRS | 1.109 | 0.267 | -0.028 | -0.077 – 0.021 |
|  | Pigmentation vs Combination | 4.042 | <0.001 | 0.058 | 0.030 – 0.087 |
|  | PRS vs Combination | 2.261 | 0.024 | 0.031 | 0.004 – 0.057 |

**Supplementary Table S3**. Polygenic risk score (PRS) distribution by both familial and sporadic patient characteristics. p-value from from Mann-Whitney test or Kruskall–Wallis test.

|  |  | **Polygenic Risk Score (PRS)** | |  |  | **Polygenic Risk Score (PRS)** | |  |
| --- | --- | --- | --- | --- | --- | --- | --- | --- |
|  | **Familial & Sporadic Patients combined** | | | | **Sporadic Patients Only** | | | |
|  |  | **Mean (SD)** | **Median (Q1, Q3)** | **p-value** |  | **Mean (SD)** | **Median (Q1, Q3)** | **p-value** |
| Sex | Male (N = 285) | 0.844 (1.042) | 0.813 (0.128, 1.496) | 0.312 | Male (N = 221) | 0.740 (1.026) | 0.717 (0.044, 1.433) | 0.661 |
|  | Female (N = 313) | 0.733 (1.179) | 0.676 (-0.043, 1.574) |  | Female (N = 234) | 0.680 (1.227) | 0.661 (-0.112, 1.477) |  |
| Fitzpatrick phototype | I + II (N = 264) | 1.083 (1.060) | 1.055 (0.403, 1.771) | <0.001 | I + II (N = 202) | 1.018 (1.086) | 1.021 (0.374, 1.676) | <0.001 |
|  | III + IV (N = 295) | 0.526 (1.104) | 0.562 (-0.203, 1.317) |  | III + IV (N = 243) | 0.460 (1.122) | 0.479 (-0.293, 1.245) |  |
| Hair color | Red (N = 61) | 1.669 (1.055) | 1.565(0.932, 2.222) |  | Red (N = 43) | 1.721 (1.069) | 1.484 (1.013, 2.239) | <0.001 |
|  | Light Blond (N = 123) | 0.987 (0.977) | 1.031 (0.370, 1.664) |  | Light Blond (N = 101) | 0.917 (0.966) | 0.980 (0.197, 1.634) |  |
|  | Blond (N = 282) | 0.620 (1.120) | 0.613 (-0.047, 1.406) |  | Blond (N = 233) | 0.544 (1.151) | 0.581 (-0.151, 1.316) |  |
|  | Brown (N = 77) | 0.364 (0.941) | 0.400 (-0.309, 1.001) |  | Brown (N = 67) | 0.312 (0.951) | 0.275 (-0.338, 0.944) |  |
| Age at diagnosis (years) | ≤ 40(N = 181) | 0.721 (1.173) | 0.7695 (-0.049, 1.446) | 0.538 | ≤ 40 (N = 162) | 0.663 (1.170) | 0.654 (-0.054, 1.345) | 0.538 |
|  | 41-64 (N = 245) | 0.807 (1.051) | 0.769 (0.125, 1.599) |  | 41-64 (N = 181) | 0.767 (1.088) | 0.708 (0.114, 1.573) |  |
|  | ≥ 65 (N = 169) | 0.814 (1.141) | 0.737 (-0.036 1.511) |  | ≥ 65 (N = 112) | 0.683 (1.160) | 0.617 (-0.287, 1.440) |  |
| No. of melanomas | 1 (N = 345) | 0.756 (1.096) | 0.715 (-0.036, 1.518) | 0.079 | 1 (N = 243) | 0.621 (1.095) | 0.618  (-0.143, 1.382) | 0.044 |
|  | 2-3 (N = 214) | 0.747 (1.096) | 0.704 (0.040, 1.463) |  | 2-3 (N = 186) | 0.760 (1.176) | 0.848 (0.046, 1.473) |  |
|  | ≥4 (N = 36) | 1.245 (1.087) | 0.999 (0.608, 2.158) |  | ≥4 (N = 26) | 1.171 (1.086) | 0.999 (0.328, 2.073) |  |
| Family history of melanoma | Yes (N = 144) | 1.032 (1.023) | 0.981 (0.375, 1.733) | 0.003 | NA | | | |
|  | No (N = 455) | 0.710 (1.133) | 0.676 (−0.037, 1.449) |  |  |  |  |  |

**Supplementary Table S4**. How different family features predict sPRS (events occurring in the family, yes or no) using multiple regression analysis.

|  | **Unstandardized coefficient, B_1_** | ***p*-value** |
| --- | --- | --- |
| Numbers of melanomas diagnosed in the family (n) | 0.156 | 0.003 |
| Multiple primary melanomas in family, yes/no | -0.204 | 0.141 |
| Melanoma diagnosed before 40 yrs in family, yes/no | 0.054 | 0.621 |
| Deaths due to melanoma in family, yes/no | -0.176 | 0.450 |
| Non-melanoma skin cancers in family, yes/no | - 0.063 | 0.664 |
| Other non-skin cancers in family, yes/no | -0.330 | 0.765 |

**Supplementary Table S5**. Standardized polygenic risk score (sPRS) distribution in dependent on number of melanoma cases in a family. P-values from Kruskall–Wallis test on independent samples.

|  |  | **Polygenic Risk Score (PRS)** | |  |
| --- | --- | --- | --- | --- |
|  | **Number of patients** | **Mean (SD)** | **Median (Q1, Q3)** | **p-value** |
| 2 cases /family | N = 57 | 0.834 (1.102) | 0.715 (-0.020, 1.700) | 0.034 |
| 3 cases /family | N = 46 | 0.949 (0.993) | 0.720 (0.289, 1.885) |  |
| 4-5 cases /family | N = 33 | 1.185 (0.838) | 1.159 (0.501, 1.560) |  |
| ≥ 6 cases /family | N = 11 | 1.810 (0.986) | 1.765 (0.977, 2.781) |  |

**Supplementary Table S6**. Standardized polygenic risk score (sPRS) distribution in patient and control individuals (combined) by characteristics. P-values from Mann-Whitney test or Kruskall–Wallis test.

|  |  | **Polygenic Risk Score (PRS)** | |  |
| --- | --- | --- | --- | --- |
|  |  | **Mean (SD)** | **Median (Q1, Q3)** | **p-value** |
| Sex | F (N = 406) | 0.515 (1.258) | 0.494 (−0.235, 1.343) | 0.18 |
|  | M (N = 372) | 0.647 (1.107) | 0.619 (-0.069, 1.399) |  |
| Hair color | Red (N = 71) | 1.585 (1.084) | 1.484 (0.810, 2.206) | <0.001 |
|  | Light blond (N = 150) | 0.884 (1.022) | 0.927 (0.121, 1.652) |  |
|  | Medium blond (N = 372) | 0.417 (1.187) | 0.449 (-0.577, 0.702) |  |
|  | Brown (N = 130) | 0.042 (1.024) | 0.074 (-0.577, 0.702) |  |
| Fitzpatrick phototype | I–II (N = 293) | 1.047 (1.050) | 1.013 (0.378, 1.742) | <0.001 |
|  | III–IV (N = 446) | 0.256 (1.180) | 0.160 (−0.418, 1.061) |  |
